# Xeroderma Pigmentosum With Ocular Surface Squamous Neoplasia: A Case Report

**DOI:** 10.7759/cureus.53204

**Published:** 2024-01-29

**Authors:** Sravanthi Malikireddy

**Affiliations:** 1 Ophthalmology, Modern Eye Hospital, Chittoor, IND

**Keywords:** basal cell carcinoma (bcc), imiquimod, interferon therapy, ocular surface squamous neoplasia (ossn), xeroderma pigmentosum

## Abstract

Xeroderma pigmentosum (XP) is a rare, autosomal recessive disorder characterized by defective DNA repair. Patients with this disorder are at increased risk of developing various oculocutaneous malignancies.

We report a rare case of a child with XP with bilateral ocular surface squamous neoplasia (OSSN) and left eye (OS) medial canthal basal cell carcinoma (BCC). Ultrasound biomicroscopy and contrast-enhanced computed tomography (CECT) of the orbit showed intraocular extension with no orbital involvement. The patient was started on topical interferon therapy in both eyes for OSSN. Topical 5% imiquimod was started for BCC for eight hours a day and then washed off. During follow-up, there was no recurrence of tumors. Since ocular and cutaneous neoplasms tend to occur at an early age in XP with a high rate of recurrence and they tend to be more aggressive, multimodal therapy with long-term follow-up is more advantageous for these patients. Topical 5% imiquimod can be used as a treatment for periocular BCC as an alternative to excision.

## Introduction

XP is an autosomal recessive disorder in which a patient has defective nucleotide excision repair to damage caused by UV radiation [1-3]. It equally affects both genders with an incidence of 1:250,000 [4,5]. It is more common in Japanese people than in others [5]. It is due to a mutation in nucleotide excision repair that causes accumulation of damaged DNA leading to increased epithelial cell turnover leading to flaky skin, pigmentation, and increased risk of ocular and cutaneous neoplasia like basal cell carcinoma (BCC) and squamous cell carcinoma (SCC) [4,5]. There are seven types and one variant form. Squamous cell carcinoma is the most common ocular and cutaneous neoplasm in XP [4,5].

Lee and Hirst have given the term ocular surface squamous neoplasia (OSSN) [6,7]. It includes the entire spectrum of dysplastic and carcinomatous lesions of the ocular surface. Later conjunctival and corneal intraepithelial neoplasms were included [7]. Dysplasia of the ocular surface can be divided into mild, moderate, and severe based on the extent of dysplastic cell invasion in the epithelium. Mild dysplasia is when the basal one-third epithelium is involved, moderate dysplasia is when the basal two-thirds is involved, and severe or carcinoma in situ is when the whole thickness is involved. Invasive carcinoma is when dysplastic cells breach the basement membrane. Risk factors include the location of a person with respect to the equator. The age of onset was younger at latitudes less than 30 degrees from the equator as compared with the age of onset at 45 degrees from the equator [6,7]. The three most important risk factors are ultraviolet B irradiation, human papillomavirus, and human immunodeficiency virus. Others are cigarette smoking, petroleum products, vitamin A deficiency, chemicals like trifluridine, arsenicals, beryllium, and hypopigmentation of the hair and eyes [8]. Following surgical excision, recurrence rates for preinvasive and invasive OSSN range from 15% to 52% [6,7].

There is no standard protocol for the treatment of OSSN. It is mainly based on preferred clinical practice. Treatment options include surgery (wide local excision) and medical management (0.04% mitomycin, interferon alpha 2b, 1% 5-fluorouracil) [8]. A comparative study of interferon and mitomycin has shown that both are equally effective in the resolution of OSSN [8]. Ten-year results show that interferon is highly effective in the treatment of OSSN <3 clock hours and as an adjunctive therapy >3 clock hours [8,9].

## Case presentation

We present a case of a 10-year-old male child who was diagnosed with XP at the age of 4 years. He came with complaints of a mass in both eyes since August 2020. The mass was first visualized in the right eye, which gradually increased in size followed by the development of a mass in the left eye eight months later. The mass started on the medial palpebral conjunctiva of both eyes then gradually involved the entire cornea with a gradual diminution of vision in the right eye and involving 2 mm of the cornea in the left eye. There was no history of pain or redness.

He had a history of excision of the mass over the left nasal ala with skin grafting on March 9, 2021, elsewhere. Two months later, he developed a recurrence in the same area and underwent wide excision with a forehead rotational flap. Biopsy was confirmatory of moderately differentiated squamous cell carcinoma.

On general examination, there were multiple pigmented lesions all over the body with areas of hypopigmentation and desquamation. On ocular examination, the patient was extremely photophobic with no perception of light in the right eye. His vision was 20/20 in the left eye. A telecanthus of 4 mm was present. Two papulonodular lesions of 0.3 mm and 0.5 mm over the medial canthal area, with pearly rolled edges, were present with surrounding crusting with distinct margins. Lower lid ectropion with keratinization of lid margins was present in both eyes.

**Figure 1:**
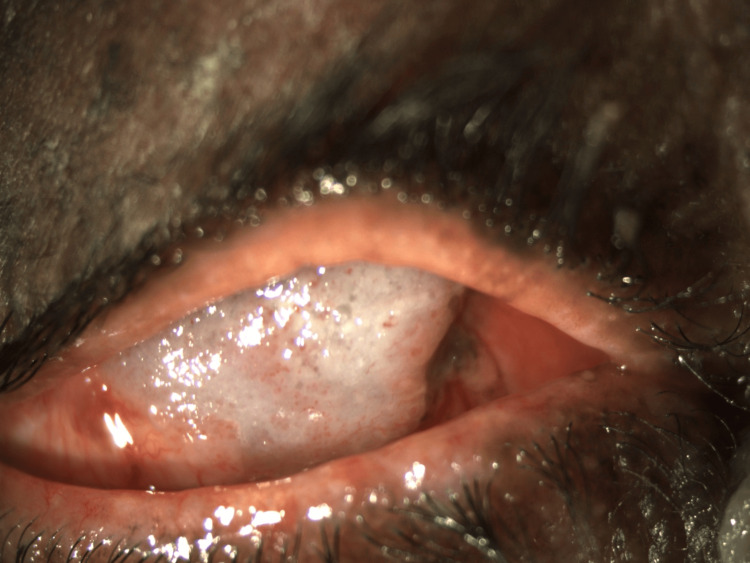
Right eye OSSN OSSN: ocular surface squamous neoplasia

The right eye (Figure 1) showed a mass extending from the medial canthus to the cornea, covering it completely. The mass had multiple pinkish spots and was of the size 13 mm in both horizontal and vertical dimensions with dilated feeder vessels. The rest of the anterior and posterior segment details were not visible.

**Figure 2:**
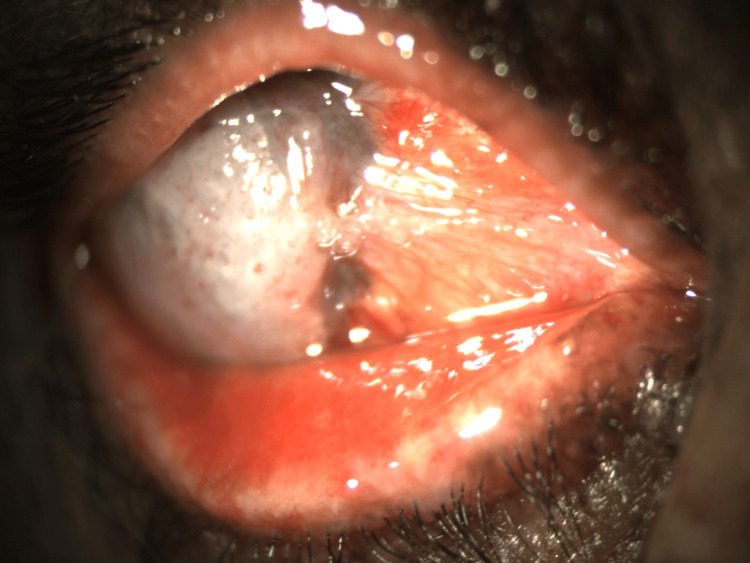
Left eye OSSN OSSN: ocular surface squamous neoplasia

The left eye (Figure 2) showed a similar mass of 3.5 mm horizontally and 5.5 mm vertically in the medial aspect of the eye, encroaching 2 mm into the cornea with dilated vessels. The anterior chamber showed no cells. The fundus of OS was normal. Intraocular pressures in both eyes were within normal limits.

Impression cytology showed dysplastic cells with inflammatory exudates. Contrast-enhanced computed tomography (CECT) head and orbit showed a nonspecific, plaque-like soft tissue mass on the surface of both globes, especially the superonasal quadrants. No post-septal extension existed. Ultrasonography of the right eye was anechoic. Ultrasound biomicroscopy showed intraocular extension into the anterior chamber. Visually evoked response (VER) showed an increased latency in the right eye.

Therefore, a provisional diagnosis of right eye OSSN with intraocular extension with left eye OSSN extending 3 clock hours with medial canthal BCC was made. Right eye enucleation, with primary implant and left eye excision biopsy and triple freeze-thaw with cryotherapy of the conjunctival margins with an amniotic membrane graft and incision biopsy of the medial canthal mass was done.

**Figure 3:**
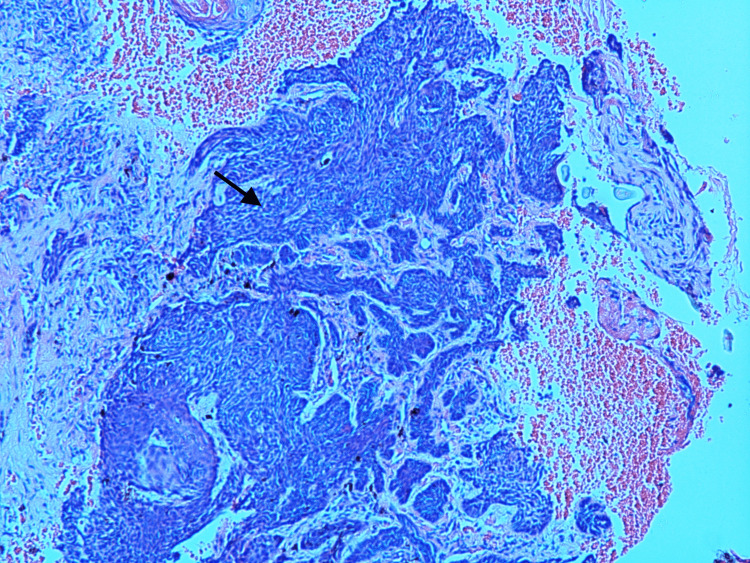
Histopathology of the medial canthal mass showed BCC with a peripheral palisade of basaloid cells BCC: basal cell carcinoma

Histopathology showed multiple, variably sized, and rounded nodules. The nodules of the tumor showed a peripheral palisade of basaloid cells at their interface with the stroma (Figure 3).

**Figure 4:**
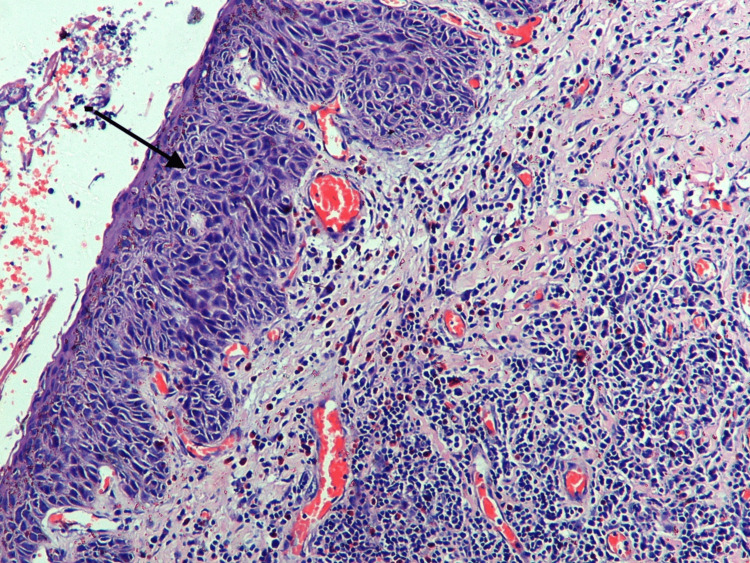
Histopathology of the conjunctival mass showing dysplastic cells involving the whole thickness of the epithelium, suggestive of OSSN OSSN: ocular surface squamous neoplasia

Histopathology of the conjunctival mass showed dysplastic cells involving the whole thickness of the epithelium, suggestive of OSSN (Figure 4). The margins free of tumor and the medial canthal mass were suggestive of BCC. The patient developed an infection over the medial canthus, which was controlled with topical and systemic antibiotics.

The patient was started on topical interferon 1 million IU/ml thrice daily in the left eye and 5% imiquimod was applied for 8 hours and washed off thrice a week in the left medial canthal area. The three-month follow-up showed no recurrence and the patient was doing well after which he was lost to follow-up.

## Discussion

We report a case of XP in a 10-year-old child with multiple oculocutaneous malignancies at the time of presentation, with a history of multiple surgeries for recurrences of skin neoplasms.

Malignancies are 1000 times more common in XP than in the general population. The mean age of presentation is 8 years compared to 60 years in the general population. Various case series have shown that the incidence of malignant ocular and cutaneous neoplasms is 40-60%. Squamous cell carcinoma is the most common of them all [9,10].

Our patient presented with OSSN in both eyes with the right eye showing intraocular extension. We did a wide local excision of OSSN in the right eye with topical interferon alfa 2b therapy to prevent recurrence while the right eye had to be enucleated.

The topical treatments that can be used in the treatment or neoadjuvant therapy of OSSN are interferon-α2b (IFN), 5-fluorouracil (5FU), and mitomycin-C (MMC) [11]. All three have similar resolution and recurrence rates but differ in cost and side-effect profile. 5-fluorouracil is the cheapest, followed by MMC. Interferon-α2b has the lowest side-effect profile followed by 5-fluorouracil, whereas mitomycin has greater surface toxicity compared to other topical therapies of OSSN due to non-cell cycle-dependent damage [11]. Its main complications include limbal stem cell deficiency, punctal stenosis, recurrent corneal erosions, and punctate keratopathy [11]. 5-fluorouracil has a cell cycle-dependent toxicity. Side effects are mild and transient, including punctate epithelial erosions and ulceration, lid toxicity, and keratoconjunctivitis [11]. Topical IFN is well-tolerated with minimal side effects such as conjunctival hyperemia, follicular conjunctivitis, corneal erosion, and epithelial microcysts. The main issue of topical therapy is compliance due to the longer duration of treatment [11,12].

We preferred interferon over mitomycin and 5-fluorouracil, as it has shown regression of the tumor with the least ocular side effects.

For BCC of the left medial canthus, a conservative approach with topical imiquimod was used, as the patient has already undergone multiple surgeries and high chance of new tumors. The other drug that can be used is 5% 5-FU cream. Both treatments are equally effective for low-risk, small superficial BCC (<2 cm in diameter) [12-14].

Imiquimod is an imidazoquinoline and acts as an immunomodulator; it has been shown to have potent anti-viral and anti-tumor activity. It has both direct and indirect mechanisms of action. Direct action is by activating caspases, resulting in apoptosis of cells and indirect action is by the release of various cytokines like IL-12, tumor necrosis factor-alpha (TNF-alpha), and interferon (INF)-gamma, which in turn increases the levels of cytotoxic T-cells and natural killer cells and stimulates anti-tumor T-cells [13]. The most common adverse effects are itching, erythema, and crusting, which usually subside with the cessation of therapy [13].

There is not much literature on the use of imiquimod in periocular tumors. Few case reports have shown that imiquimod successfully treated and prevented the recurrence of tumors in XP but there is no long-term study to back this up [10,15].

XP is associated with the occurrence of multiple tumors at a time so a surgical approach is not always feasible in these patients. Topical chemotherapy may be a good option in this scenario; however, a randomized controlled study for the evaluation of the use of topical chemotherapy in periocular tumors may provide stronger evidence of its use in these cases.

## Conclusions

XP is a rare, autosomal, recessive disorder. Ocular and cutaneous neoplasms tend to occur at an early age in patients with XP. They tend to have a high rate of recurrences and are more aggressive. Therefore, multimodal therapy is more useful than surgical treatment alone, with follow-up. Strict sun-protective measures like sunscreen, the use of eye shields, and regular follow-ups are required to detect malignancies at the earliest. Various studies have shown that topical chemotherapy can be used in selected cases of low-risk BCC with a size <2 cm in diameter. Complete excision of tumors like Mohs micrographic surgery remains the gold standard with a superior treatment outcome in most of the cases. Since our case was an exception, as the patient underwent repeated surgeries and the tumor itself was less than 2 cm, we preferred topical therapy. Topical 5% imiquimod can be used as a treatment for periocular BCC as an alternative to excision.
